# Commentary: Effect of gluten-free diet on autoimmune thyroiditis progression in patients with no symptoms or histology of celiac disease: a meta-analysis

**DOI:** 10.3389/fendo.2024.1459941

**Published:** 2024-08-22

**Authors:** Edilene Maria Queiroz Araújo, Helton Estrela Ramos, Virginia Fernandes Moça Trevisani

**Affiliations:** ^1^ Department of Life Sciences, State University of Bahia, Bahia, Brazil; ^2^ Postgraduate Program in Evidence Based Medicine, Federal University of São Paulo, São Paulo, Brazil; ^3^ Biorregulation Department, Institute of Health Sciences, Federal University of Bahia, Bahia, Brazil

**Keywords:** gluten-free diet, Hashimoto’s thyroiditi, non-celiac disease gluten sensitivity, metanalysis, letter to editor

We carefully assessed the meta-analysis by Piticchio et al., 2023, which aimed to “examine all available data in the literature on the effect of a gluten-free diet (GFD) on TgAb, TPOAb, TSH, FT4 and FT3 levels in patients with TH and without symptoms or histology of celiac disease (CD)”. However, we noted some concerns about the quality of this systematic review, which we hope merit consideration.

A systematic literature review is a secondary study that brings together similar studies, published or not, and critically evaluates their methodology. It can gather quantitative results in a statistical analysis or meta-analysis, when possible. It is considered the best level of evidence for making decisions on therapeutic issues, as it synthesizes similar primary studies of good quality (1, 2). To avoid analysis bias in a systematic review, the methods for selecting and analyzing the data are established before the review is carried out, in a rigorous and well-defined process.

Firstly, the authors did not define the methods in a rigorous and well-defined process, at least in terms of protocol registration, as they did not present a previous registration number on any platform. Drawing up a protocol allows the systematic review to be organized, more transparent, and with a lower risk of bias (1). Another issue observed was that Piticchio et al., 2023 reported that the publication was only a meta-analysis as explained in the title, but in the first line of 2.1 Construction of the review under Materials and Methods, the authors stated that it was a systematic review (SR). There is a difference between carrying out just a meta-analysis and an SR with meta-analysis. A meta-analysis is not carried out in all cases; it should only be carried out if the studies included are similar, i.e., if the sample, the types of studies, the intervention, and the clinical results are homogeneous (1). In this case, as will be seen below, the studies were not similar, which would require subgrouping.

Secondly, the authors of the publication stated that they carried out the meta-analysis according to the Meta-analysis of Observational Studies in Epidemiology (MOOSE) and through the National Heart, Lung, and Blood Institute Quality Assessment Tool for Observational Studies; the analyses can be seen in Supplementary Figure 1. If the included articles carried out an intervention, gluten-free diet (GFD), they could not be classified as observational articles. So, why did the authors not use more appropriate assessment tools, such as ‘Risk of Bias in Non-Randomized Studies of Interventions’ (Rob 1), ‘risk-of-bias tool for randomized trials’ (Rob 2) (1), the Jadad Scale (2), and/or A Measurement Tool to Assess Systematic Reviews (3)? It is possible to see that important issues to be analyzed in clinical trials were not addressed in the MOOSE tool used, as shown in Supplementary Figure 1.

Moreover, there was also a lack of tools to help with the writing of the systematic review, such as The Preferred Reporting Items for Systematic Reviews and Meta-Analyses (PRISMA) (4). The PRISMA guidelines are designed to ensure transparency and rigor in the reporting of systematic reviews. In the topic of the Introduction section, for example, there was no mention of the subject of gluten and the Gluten-free diet (GFD). The authors also failed to mention whether they followed an acronym, such as PICO, when drawing up the eligibility criteria, objectives, research question, and research strategy (1, 4). The lack of this acronym may have led to the absence of some points: clear objectives in the article, since in the conclusions the authors talked about inflammation, which was not mentioned previously, and did not state how they assessed this outcome. Regarding the eligibility criteria, there was no information on the diet, whether the diet would be totally gluten-free or whether it could be gluten-reduced, and there was no information on which diets would be compared to the gluten-free diet, i.e., specifying the comparator group. In one of the articles included in this meta-analysis, there were multiple interventions, GFD (gluten-free diet) and lifestyle (5). As such, this article needed to be carefully assessed or excluded; it should also be added that there was no mention of which types of studies would be included and excluded in this meta-analysis.

As for the choice of database, only two were searched, PubMed and Scopus, which may have restricted the number of articles found and the number of people in the sample. Furthermore, the search language was restricted to English only. Given the availability of resources for translating articles into other languages, excluding non-English articles from the search does not seem justified. Finally, the authors described that some stages were carried out by two reviewers (item 2.5), sometimes independently (item 2.4), but at no point did they state that the procedure was carried out blindly, as indicated in the main methodological guidelines for preparing systematic reviews (2).

At the end of the meta-analysis, the authors concluded that the evidence was not sufficient to recommend GFD for all patients diagnosed with Hashimoto’s thyroiditis (HT). However, it is not up to the systematic review to “recommend”, but rather to gather scientific evidence to support the specialist’s decision. In addition the authors also did not assess the certainty of the evidence using the GRADE methodology approach by two independent reviewers. This is an extremely important stage that takes place after the meta-analysis has been carried out. Finally, our research group considered that the research lacks robustness and that there are methodological flaws that need to be readjusted, in addition to revising the eligibility criteria, so that we can answer and fully address the question of whether or not GFD is effective in treating HT in non-celiac disease.

Lastly, we would like to cordially thank the editors for allowing us to clarify these points.
